# SNP-Based QTL Mapping of 15 Complex Traits in Barley under Rain-Fed and Well-Watered Conditions by a Mixed Modeling Approach

**DOI:** 10.3389/fpls.2016.00909

**Published:** 2016-06-27

**Authors:** Freddy Mora, Yerko A. Quitral, Ivan Matus, Joanne Russell, Robbie Waugh, Alejandro del Pozo

**Affiliations:** ^1^Instituto de Ciencias Biológicas, Área de Biología Molecular y Biotecnología, Universidad de TalcaTalca, Chile; ^2^Centro de Mejoramiento Genético y Fenómica Vegetal, Facultad de Ciencias Agrarias, PIEI Adaptación de la Agricultura al Cambio Climático (A2C2), Universidad de TalcaTalca, Chile; ^3^Centro Regional de Investigación Quilamapu, Instituto de Investigaciones AgropecuariasChillán, Chile; ^4^The James Hutton InstituteDundee, Scotland

**Keywords:** drought, marker segregation distortion, RCSL, physiological trait, kinship

## Abstract

This study identified single nucleotide polymorphism (SNP) markers associated with 15 complex traits in a breeding population of barley (*Hordeum vulgare* L.) consisting of 137 recombinant chromosome substitution lines (RCSL), evaluated under contrasting water availability conditions in the Mediterranean climatic region of central Chile. Given that markers showed a very strong segregation distortion, a quantitative trait locus/loci (QTL) mapping mixed model was used to account for the heterogeneity in genetic relatedness between genotypes. Fifty-seven QTL were detected under rain-fed conditions, which accounted for 5–22% of the phenotypic variation. In full irrigation conditions, 84 SNPs were significantly associated with the traits studied, explaining 5–35% of phenotypic variation. Most of the QTL were co-localized on chromosomes 2H and 3H. Environment-specific genomic regions were detected for 12 of the 15 traits scored. Although most QTL-trait associations were environment and trait specific, some important and stable associations were also detected. In full irrigation conditions, a relatively major genomic region was found underlying hectoliter weight (HW), on chromosome 1H, which explained between 27% (SNP 2711-234) and 35% (SNP 1923-265) of the phenotypic variation. Interestingly, the locus 1923-265 was also detected for grain yield at both environmental conditions, accounting for 9 and 18%, in the rain-fed and irrigation conditions, respectively. Analysis of QTL in this breeding population identified significant genomic regions that can be used for marker-assisted selection (MAS) of barley in areas where drought is a significant constraint.

## Introduction

Abiotic stresses can significantly reduce crop yields and restrict the latitudes and soils on which commercially important species can be cultivated (Lobell and Field, 2007; Jacobsen et al., 2012). The development of drought-tolerant genotypes as well as genotypes with higher water-use efficiency is of global interest as populations continue to increase and water availability decreases (Araus et al., 2002, 2008; Cattivelli et al., 2008). In barley (*Hordeum vulgare* L.), a large number of morphological and physiological traits are linked to drought tolerance (Chen et al., 2010; del Pozo et al., 2012) which exhibit strong environmental interactions (Tondelli et al., 2006). Increasing tolerance to drought stress has become a major goal for barley breeding programs particularly in light of prolonged drought periods as a result of climate change (Wehner et al., 2015).

To determine the genetic basis of complex traits, important genetic, and genomic resources have been developed in a wide range of species (Kota et al., 2003; Mora et al., 2015), including barley (Kota et al., 2003; Close et al., 2009; Zhou et al., 2015). Single nucleotide polymorphism (SNP) markers are the most abundant sequence variations encountered in eukaryotic genomes (Griffin and Smith, 2000), and in barley offer the potential for generating very high-density genetic maps (Close et al., 2009), providing a useful tool for quantitative trait locus/loci (QTL) mapping for marker-assisted selection (MAS; Sato and Takeda, 2009; Szűcs et al., 2009).

Conventionally, quantitative trait locus/loci (QTL) mapping is carried out using markers that follow a Mendelian segregation ratio, which depends on the population under investigation (Xu, 2008). The phenomenon that alleles at a locus deviate from the Mendelian expectation has been defined as segregation distortion (Zhang et al., 2010), which has been encountered in many commercially important species, such as maize (Lu et al., 2002), rice (Xu et al., 1997), tomato (Paterson et al., 1988), and barley (Malosetti et al., 2011). Biologically, segregation distortion can be due to chromosome loss, genetic isolation mechanisms, and the presence of viability genes. Non-biological factors such as scoring errors and sampling errors also can contribute to segregation distortion (Alheit et al., 2011).

Construction of genetic maps and QTL analysis using distorted markers is risky because the basic assumption of Mendelian segregation is violated, and, consequently most of these markers are removed from the subsequent QTL analyses (Luo et al., 2005; Xu, 2008). Moreover, several authors found that distorted markers influence the estimation of genetic intervals and the markers order on a same chromosome (Lorieux et al., 1995a,b; Zhu et al., 2007). Because these markers are routinely removed, valuable information around these regions is lost (Luo et al., 2005). However, if distorted markers are handled properly, their effects on genetic map construction and QTL identification and mapping can be significantly improved (Xu, 2008; Alheit et al., 2011; Hashemi et al., 2015).

From an analytical standpoint, many QTL mapping procedures have been developed for Mendelian populations, but few are available for markers that do not segregate in a typical Mendelian fashion (Zhu et al., 2007; Xu, 2008). Recently, Malosetti et al. (2011) found severe allele frequency distortions in many chromosomal regions in a RIL population of barley. The authors stated that violation of the basic assumptions implies that the genetic covariance between genotypes (i.e., genetic relatedness) in the population is not homogeneous, and analogous to association mapping studies, a QTL mapping mixed model that account for this heterogeneity should be used to avoid false QTL detection. In addition, mixed models have been used to investigate QTL-by-environment interaction (Malosetti et al., 2004; Boer et al., 2007) and to map QTL for several traits simultaneously (Malosetti et al., 2008).

In barley, a large number of mapping populations have been developed to map QTL. Further, advanced mapping populations, including near-isogenic lines (NILs), chromosome segment substitution lines (CSSLs), and recombinant chromosome substitution lines (RCSLs, Schmalenbach et al., 2008, 2011; Sato and Takeda, 2009; Naz et al., 2012), have also been developed to facilitate the genetic dissection of complex traits. As a consequence, many QTL controlling complex traits including agronomic, and morphological traits, yield component, disease resistance, tolerance to abiotic stress, and malting quality have been identified (Pillen et al., 2004; Talame et al., 2004; Li et al., 2005; Gyenis et al., 2007). However, studies that have identified QTL for drought-related morphological and physiological traits are still scarce in barley (Chen et al., 2010; Mir et al., 2012; Sayed et al., 2012; Kalladan et al., 2013; Li et al., 2013; Wójcik-Jagła et al., 2013; Honsdorf et al., 2014; Mansour et al., 2014; Naz et al., 2014). Thus, a breeding program has been developed in the Mediterranean area of central Chile, in areas where drought is a significant constraint to yield. This study identifies SNP markers associated with 15 complex traits (including physiological and morphological traits) in a breeding population consisting of 137 RCSLs of barley (Matus et al., 2003), evaluated under contrasting water availability. Given that markers showed a very strong segregation distortion, a QTL mapping mixed model was employed to account for the heterogeneity in genetic relatedness between genotypes.

## Materials and methods

### Plant material and field evaluation

A set of 137 RCSL were evaluated in field conditions in Santa Rosa (35°78′ S, 72°17′ W), in the Mediterranean climatic region of central Chile, under rain-fed and fully irrigated conditions in 2008–2009. The average annual temperature in this region is 13°C, the minimum average is 3°C (July), and the maximum 28.6°C (January). Monthly maximum and minimum temperatures and precipitation during 2008 are in Table S1. The annual precipitation in 2008 was 992 mm but the amount during the growing season was 120 mm. The soil is a sandy loam, Humic Haploxerand, Andisol. For fully irrigated conditions, four furrow irrigation of 50 mm water was applied from heading to maturity.

The RCSL population was developed using the advanced backcross strategy of Tanksley and Nelson (1996). The accession of *Hordeum spontaneum* (Caeserea 26-24 from Israel) was the donor parent and *Hordeum vulgare* subsp. *vulgare* “Harrington” (a North American malting quality standard) was the recurrent parent (Matus et al., 2003). The recurrent parent was used as the female and the donor as the male to obtain the F_1_ generation. Finally, the lines were obtained using two backcrosses with the recurrent parent and six generations of self-pollination (BC_2_F_6_).

The field trial was arranged in a 14 × 10 alpha-lattice design with two replications (“Harrington” cultivar was replicated more times for arrangement *v* = 14 × 10 = 140). Fertilizer and field management practices recommended for optimum barley production were used (Inostroza et al., 2009; del Pozo et al., 2012). The 15 different morphological, agronomic, and physiological traits measured during this experiment are described in Table 1.

**Table 1:** **List of 15 morphologic, agronomic, and physiological traits measured in a RCSL population of barley under two contrasting environmental conditions in southern Chile**.

**Trait**	**Abbreviations**	**Type**	**Unit**
Peduncle length	PL	Morphologic	cm
peduncle extrusion	PE	Morphologic	cm
Spike length excluding the awns	SL	Morphologic	cm
Plant height	PH	Agronomic	cm
Tillers	TN	Agronomic	Number per m^−2^
Dry weight in tillering	DWT	Agronomic	g·m^−2^
Biological yield	BY	Agronomic	g·m^−2^
Hectoliter weight	HW	Agronomic	g
Harvest index	HI	Agronomic	—
Kernel per spike	KS	Agronomic	Number
Thousand kernel weight	TKW	Agronomic	g
Grain yield	GY	Agronomic	ton·ha^−1^
Relative water content	RWC	Physiological	%
Intercept PAR	IPAR	Physiological	—
Chlorophyll fluorescence at anthesis	Fv/Fm	Physiological	—

### Phenotypic data analysis

A mixed linear modeling approach was employed for phenotypic data analysis using the MIXED procedure in SAS. Field data were analyzed on the basis of the statistical model (Stich et al., 2008):

$$\begin{array}{l} y_{ijno}=\mu +g_{i}+l_{j}+{\left(gl\right)}_{ij}+r_{nj}+b_{onj}+\varepsilon_{ijno} \end{array}$$

where *y*_*ijno*_ is the observed phenotype for the *i*th RCSL at the *j*th location in the *o*th incomplete block of the *n*th replicate, μ is an intercept term (overall population mean), *g*_*i*_ is the genotypic effect of the *i*th entry, *l*_*j*_ is the effect of the *j*th location, *r*_*nj*_ is the effect of the *n*th replicate of the *j*th location, *b*_*onj*_ is the effect of the *o*th incomplete block of the *n*th replication of the *j*th location, and ε_*ijno*_ is the residual or within-plot error. To analyze the effect of environment (rain-fed and fully irrigated) on the RCSL population, environment (contrasting environmental condition), genotype, and genotype-environment interaction were considered as fixed effects while replication and block were analyzed as random effects. Error variances were assumed to be heterogeneous among locations according to Stich et al. (2008). Adjusted entry means *M*_*i*_ were calculated for each RCSL as:

$$\begin{array}{l} M_{i}=\hat{\mu }+{ĝ}_{i} \end{array}$$

where $\hat{\mu }$ and ĝ_*i*_ are the generalized least-squares estimates of μ and *g*_*i*_, respectively. Adjusted entry means, calculated by environment and in a combined analysis, were latter used in the analysis of QTL. The CORR procedure of SAS was used to estimate Pearson correlations (*r*, *n* = 137) between pairs of traits.

### DNA extraction and genotyping

Leaf tissues were harvested from each plant and crushed with liquid nitrogen. Genomic DNA was extracted from 200 to 300 mg of leaf sample using Qiagen DNeasy Plant mini kit (QIAGEN Co.). DNA concentration was determined by nanodrop (Thermo Fisher Scientific) and adjusted to 100 ng/μl. DNA samples were sent to the Southern California Genotyping Consortium, Illumina BeadLab at the University of California, Los Angeles (UCLA) for the OPA-SNP assay with the 1536-plex detection platform of barley OPA 1 (BOPA1) developed by Close et al. (2009). SNP markers are distributed across the seven barley chromosomes. The SNP loci were designated by HarvEST:Barley unigene assembly #32 numbers (http://harvest.ucr.edu/). Genotyping of the 137 RCSLs and their parents was performed by Illumina GoldenGate assay. The order of polymorphic markers from BOPA1 was performed using MEGA5 software (Tamura et al., 2011). Moreover, the chromosome segments introgressed into *H. vulgare* cv. Harrington from *Hordeum spontaneum* were estimated from the graphical haplotypes (Van Berloo et al., 2008) in each of the recombinant lines selected.

### QTL mapping by a mixed modeling approach

For the segregation data of each SNP marker, deviations from the Mendelian ratios (1:1 ratio for RSCL populations) were tested using the Chi-square test. Given that SNP markers did not segregate in an expected Mendelian ratio (details in the Results Section) a mixed model (Stich et al., 2008) was employed for QTL analysis of a designed cross using a structured variance–covariance matrix, where the structure was induced by selection (Malosetti et al., 2011). This QTL mapping mixed model accounts for the heterogeneity in genetic relatedness between genotypes. The hypothesis of association of SNP markers with the 15 target traits was tested using the following mixed model implemented in the program TASSEL 3.0 (Bradbury et al., 2007):

(1) $$\begin{array}{l} M_{i}=\mu_{1}+x_{i}\alpha +u_{i}+e_{i} \end{array}$$

where *M*_*i*_ is the adjusted entry mean of the *i*th RCSL, μ_1_ is an intercept term, *x*_*i*_ is the SNP genotype of *i*th RCSL, α is the additive allele substitution effect (SNP effect), *u*_*i*_ is the residual genetic background effect of the *i*th entry, and *e*_*i*_ is the random residual effect. *u*_*i*_ is assumed to follow a normal distribution with variance-covariance matrix G $=\sigma_{u}^{2}\cdot 2\cdot$ K, where K is the coefficient of co-ancestry matrix between entries. The threshold used for declaring an association significant was *P* < 0.01. According to Malosetti et al. (2011) this mixed modeling approach for QTL detection can accommodate the extra genetic covariance by embedding kinship information in the model, leading to appropriate tests, and minimizing the rate of false QTL or gene detection. Variance components were estimated using the Restricted Maximum Likelihood (REML) method. Additionally, false discovery rate (FDR)-adjusted *p*-values were calculated using PROC MULTTEST in SAS software. The Bayesian information criterion (BIC) was used to compare the simple model that ignore kinship information and the model with a structured variance–covariance matrix (kinship information).

## Results

### Phenotypic data analysis

The statistical analysis of fixed effects for the 15 complex traits under study are summarized in Table 2. According to the *F*-values (type III tests of fixed effects), the 137 RCSL presented significant differences at *P* < 0.01 in most traits under study. Significant differences were observed between both contrasting water regimes for most traits, including biological yield (BY) and grain yield (GY). In fact, the average GY under rain-fed was reduced by 81% in relation to fully irrigated condition, indicating that the barley RCSL lines were exposed to a severe water stress. Also, there was a strong reduction in BY (−59.9%), dry weight at tillering (DWT; −39.9%), tiller number (TN; −48.1%) and thousand-kernel weight (TKW; −28.1%) (Table 2). Peduncle length (PL), peduncle extrusion (PE), plant height (PH), and the physiological trait IPAR also evidenced significant environmental effect. There were significant interactions between RCSL and environment for PE, PH, TN, hectoliter weight (HW), kernels per spike (KS), and GY.

**Table 2:** **Summary of statistical analysis of fixed effects for 15 complex traits measured in a RCSL population of barley under rain-fed and well-watered conditions**.

**Trait**	**Environmental condition**		**Environment effect**	**GEI**	**RCSL**
	**Rain-fed**	**Fully irrigated**			
**MORPHOLOGIC**					
PL (cm)	17.8 (11.9–28.2)	22.8 (14.0–34.8)	^**^	NS	^**^
PE (mm)	2.7 (1.3–3.5)	2.4 (0.5–4.6)	^**^	^*^	^*^
SL (cm)	9.4 (5.6–21.6)	9.4 (6.2–12.1)	NS	NS	^**^
**AGRONOMIC**					
PH (cm)	61.9 (40–85)	83.7 (65–120)	^**^	^**^	^**^
TN (number·m^−2^)	357.1 (115–745)	688.3 (160–1235)	^**^	^*^	^**^
DWT (g·m^−2^)	121.9 (24.9–404.8)	201.0 (20.2–934.4)	^**^	NS	NS
BY (g·m^−2^)	434.6 (75–1325)	1085.8 (200–1950)	^**^	NS	^*^
HW	67.6 (51.3–73.2)	69.4 (63–9.1)	NS	^*^	NS
HI	0.3 (0.03–0.65)	0.3 (0.1–0.5)	NS	NS	^**^
KS	17.5 (7.9–28)	21.4 (10.8–29.3)	NS	^*^	^**^
TKW	40.1 (12.8–69.7)	55.8 (37.7–89.1)	^**^	NS	NS
GY (ton·ha^−1^)	1.0 (0.01–3.03)	5.3 (1.1–8.5)	^**^	^**^	^**^
**PHYSIOLOGICAL**					
RWC (%)	83.5 (52–101)	86.0 (43–127)	NS	NS	NS
IPAR	35.4 (7.5–59.9)	51.8 (14.6–79.8)	^**^	NS	^*^
Fv/Fm	0.61 (0.3–0.7)	0.65 (0.5–0.7)	NS	NS	NS

Data are presented as phenotypic means with minimum and maximum values in parentheses. GEI, genotype-environment interactions; NS, not significant.

* Significant at the 0.05 probability level.

** Significant at the 0.01 probability level.

Estimates of phenotypic correlations among traits are shown in Table 3. Grain yield was positively and significantly correlated with TN, BY, HW, HI, and KS, and negatively correlated with PL and PH (*P* < 0.01, Table 3). The physiological trait IPAR was significantly and positively correlated with biological yield (*r* = 0.6) and grain yield (*r* = 0.41). Relative water content (RWC), PE, and chlorophyll fluorescence (Fv/Fm) were not significantly correlated (*P* > 0.05) with most of the variables studied.

**Table 3:** **Pearson correlation coefficients among mean variables (adjusted entry means) measured in a RCSL population of barley under rain-fed and well-watered conditions**.

**Trait**	**PL**	**PE**	**SL**	**PH**	**TN**	**DWT**	**BY**	**HW**	**HI**	**KS**	**TKW**	**GY**	**RWC**	**IPAR**	**FV/FM**
PL	1														
PE	0.08	1													
SL	0.11	0.15	1												
PH	0.37^**^	0.19^*^	0.37^**^	1											
TN	−0.21^**^	−0.15	−0.31^**^	−0.48^**^	1										
DWT	−0.01	0.15	0.18^*^	0.12	0.16	1									
BY	−0.05	−0.10	0.23^**^	0.05	0.57^**^	0.28^**^	1								
HW	−0.21^*^	−0.08	−0.05	−0.03	0.13	0.04	0.17^*^	1							
HI	−0.12	−0.11	−0.08	−0.50^**^	0.52^**^	0.20^*^	0.40^**^	0.26^**^	1						
KS	−0.19^*^	−0.04	0.64^**^	0.15	−0.01	0.17^*^	0.47^**^	0.18^*^	0.24^**^	1					
TKW	0.22^**^	0.24^**^	0.19^*^	0.07	−0.17^*^	0.21^*^	0.06	0.07	0.20^*^	0.06	1				
GY	−0.35^**^	−0.07	0.09	−0.30^**^	0.44^**^	0.14	0.49^**^	0.48^**^	0.61^**^	0.47^**^	−0.08	1			
RWC	−0.06	0.09	−0.13	−0.17^*^	0.10	0.06	0.11	−0.08	0.14	−0.13	0.10	0.06	1		
IPAR	−0.11	−0.01	0.22^*^	0.14	0.26^**^	0.25^**^	0.60^**^	0.09	0.11	0.34^**^	−0.16	0.41^**^	0.05	1	
Fv/Fm	0.13	0.05	0.14	0.09	−0.10	−0.17^*^	−0.09	0.01	−0.09	0.07	−0.12	0.03	−0.11	−0.02	1

* Significant at the 0.05 probability level.

** Significant at the 0.01 probability level.

### Kinship matrix to avoid false-positives

**Figure 1:**
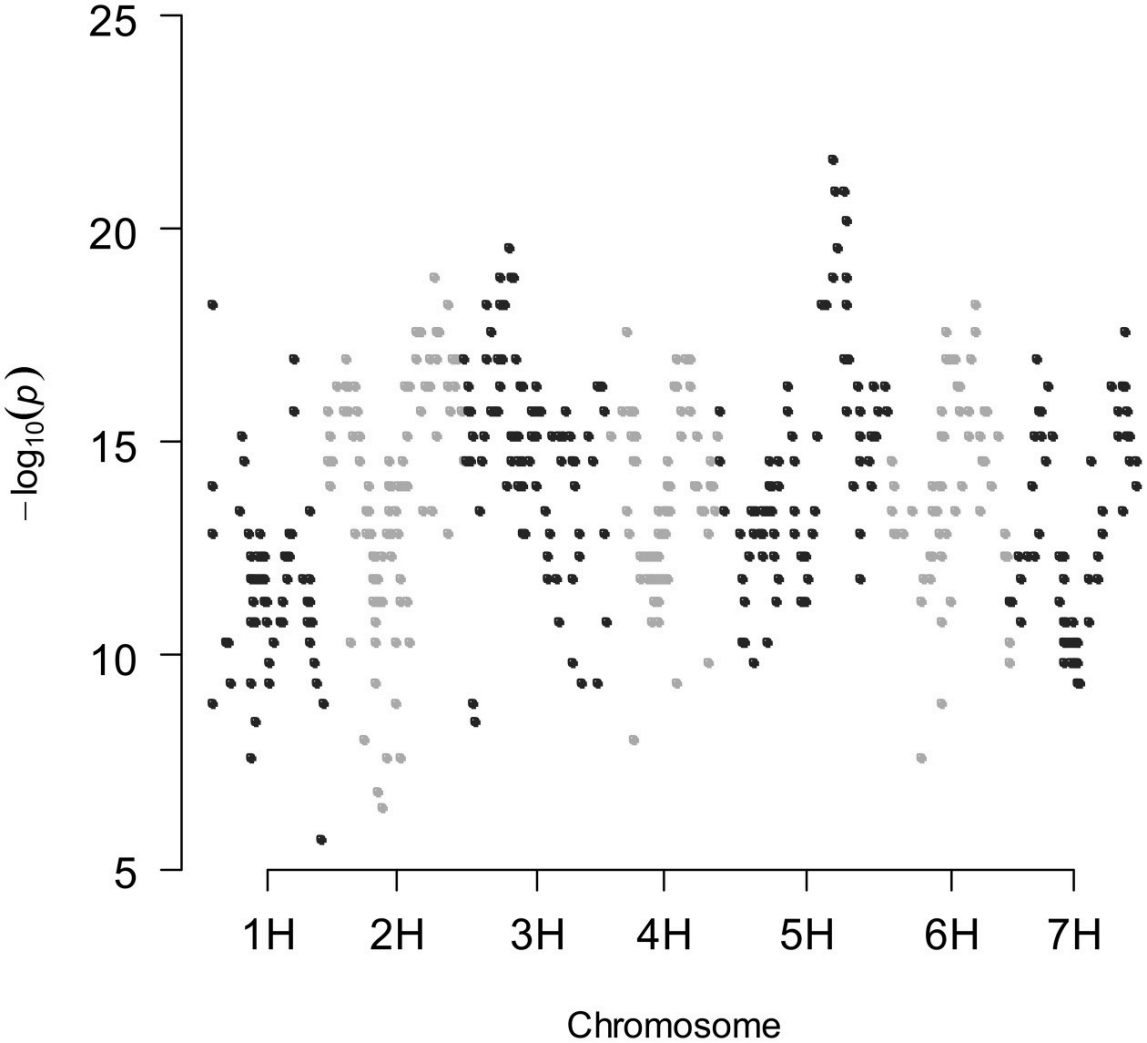
**Chi-square test for segregation distortion of SNP markers along the seven linkage groups in the RCSL population of barley**.

A large number of spurious QTLs were detected when the genetic covariance matrix is ignored in the mixed model. In fact, 444, 460, and 516 false-positives were detected for all traits studied in the rain-fed and fully irrigated conditions, and combined analysis, respectively, using a structured variance–covariance matrix in a mixed model, where the structure was induced by selection. This QTL mapping mixed model accounts for the heterogeneity in genetic relatedness between genotypes. Figure 1 shows the results of segregation distortion analysis based on the *P*-values, obtained by the chi-square test (*P*-values were plotted on a −Log10 scale). According to the Bayesian information criterion (BIC) there was significant evidence against the simple models that ignore kinship information in most of the traits studied (Table S2); between the two competing models, the best model is the one that has the smallest BIC-value. In the combined analysis, BIC was smaller for the mixed model that includes the kinship information (K model), for most of the traits. Therefore, the use of a QTL detection model that accounts for the heterogeneous genetic relatedness between RCSLs lines, caused by the uneven sharing of genetic background, is clearly necessary.

### SNP-Based QTL mapping of complex traits

**Figure 2:**
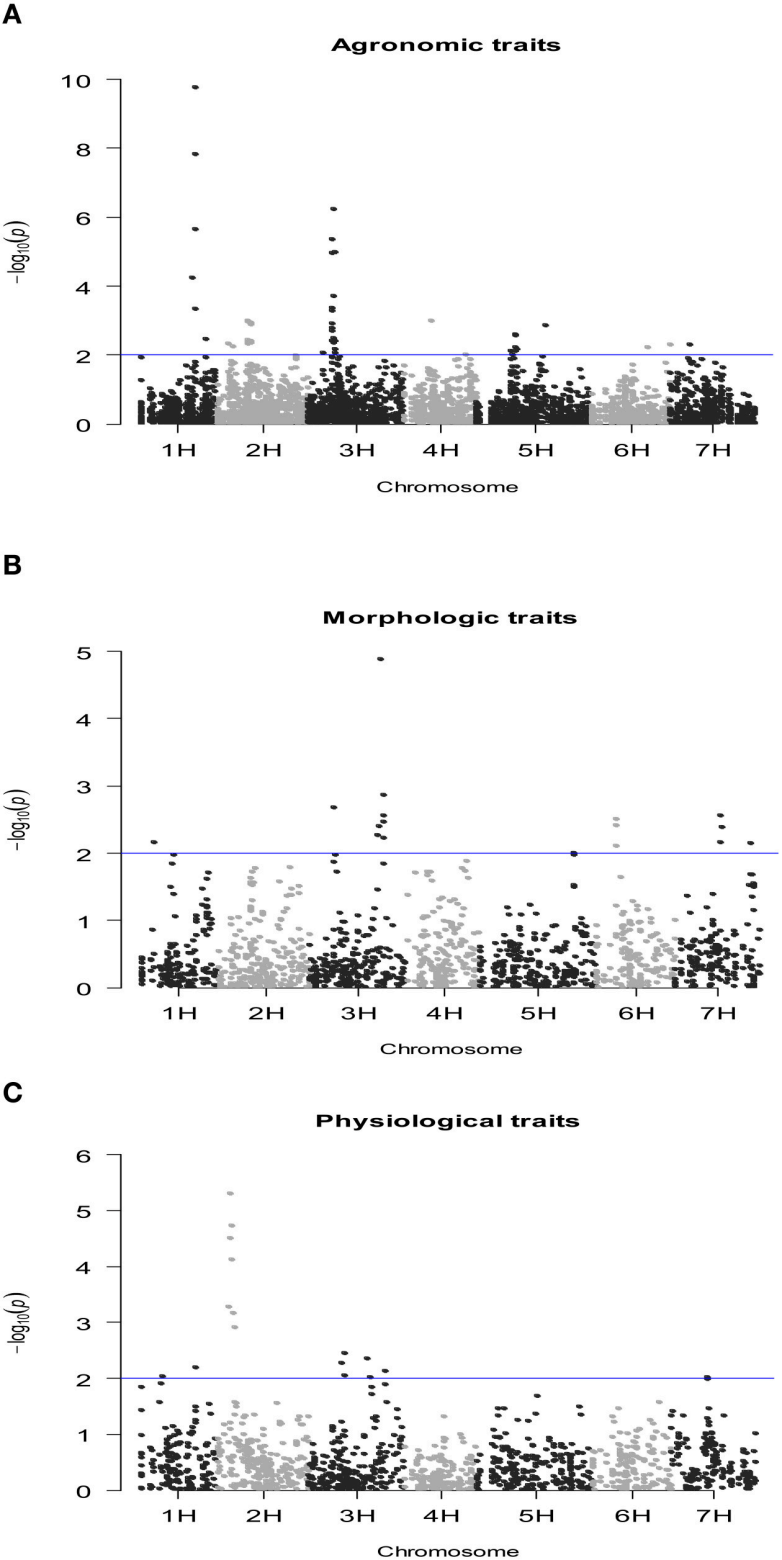
**Manhattan plot of the genome-wide QTL study in a RCSL population of barley, targeting agronomic (A), morphologic (B), and physiological (C) traits, evaluated under well-watered conditions**. The x-axis shows the chromosomes and the SNP order. The y-axis shows the −Log10(*P*-value) for each SNP marker.

**Figure 3:**
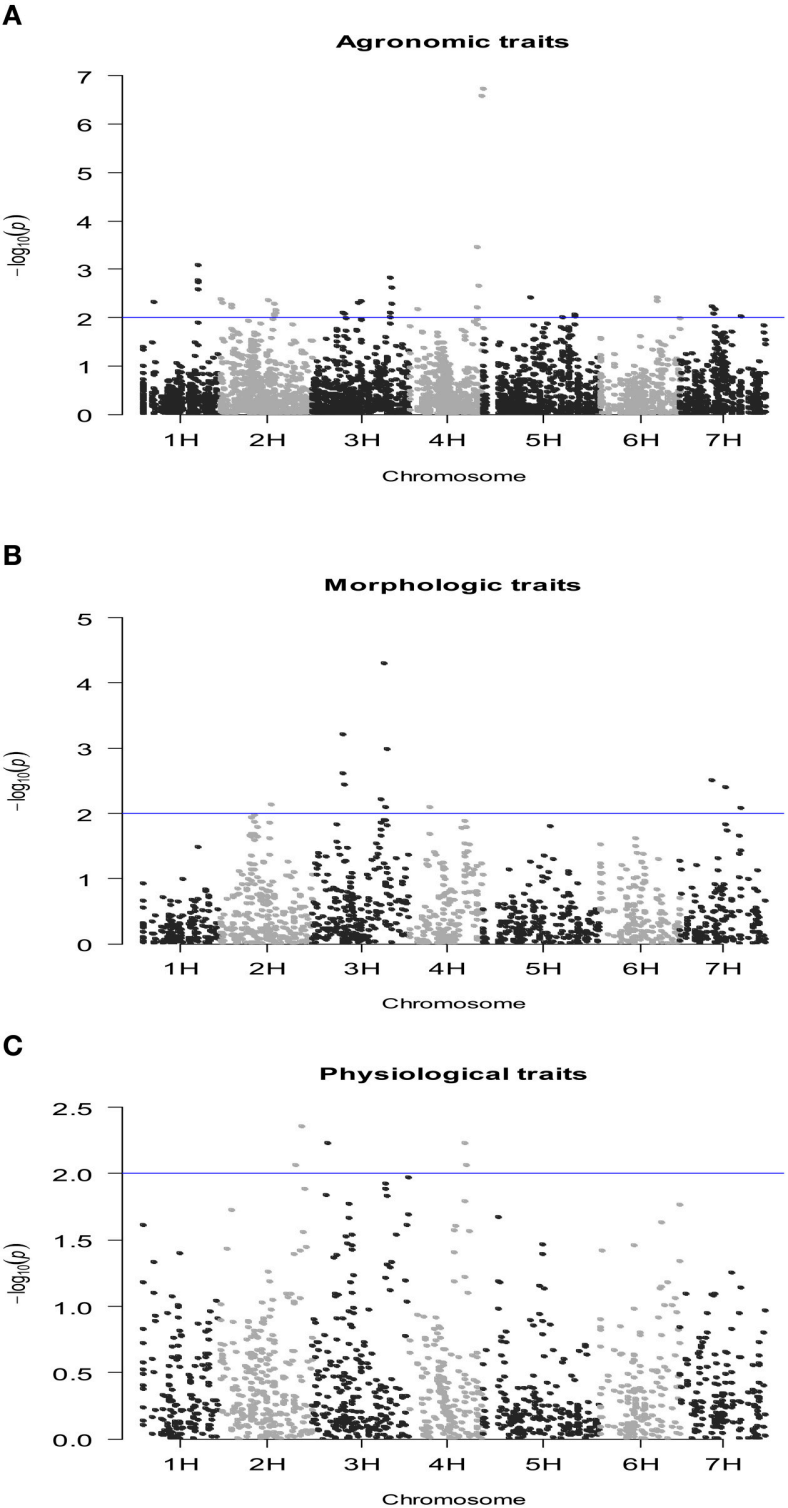
**Manhattan plot of the genome-wide QTL study in a RCSL population of barley, targeting agronomic (A), morphologic (B), and physiological (C) traits, evaluated under rain-fed conditions**. The x-axis shows the chromosomes and the SNP order. The y-axis shows the −Log10(*P*-value) for each SNP marker.

The number of QTLs detected for all the traits under study, including the chromosome number and the percentage of the total phenotypic variation explained by the QTLs, are summarized in Table 4 (details of all significant SNP–trait associations are given in Table S3). The Manhattan plots of the genome-wide QTL study showed a lower number of significant QTLs under rain-fed (Figure 2) in comparison with fully irrigated conditions (Figure 3). In fact, 57 QTLs were detected in rain-fed, which accounted for 5–22% of the phenotypic variation (KS and PH, respectively). In the fully irrigated condition, a total of 84 SNPs were significantly associated with the traits studied, which explained from 5 to 35% of phenotypic variation (TN and HW, respectively). In the combined analysis, 92 QTLs were detected to be highly significant, which accounted for 5–21% of the phenotypic variation.

**Table 4:** **Summary of QTLs detected for 15 complex traits in a RCSL population of barley evaluated under contrasting environment conditions**.

**Trait**	**Rain-fed**			**Fully irrigated plants**			**Combined**		
	**NQ**	**ChN**	**PV (%)**	**NQ**	**ChN**	**PV (%)**	**NQ**	**ChN**	**PV (%)**
**MORPHOLOGIC**									
PL	9	3H, 7H	5.3–13	11	1H, 3H, 7H	5.5–15	12	3H, 4H, 7H	5.1–18
PE	2	2H, 4H	5.3–5.5	4	6H, 7H	5.6–6.7	4	4H, 6H	5.3–6.8
SL	1	7H	6.6	2	3H, 5H	5.1–7.2	11	2H, 3H, 7H	5.1–7.5
**AGRONOMIC**									
PH	7	2H, 3H, 4H	5.8–22	7	2H, 4H, 6H	5.7–8.3	13	2H, 3H, 4H	5.1–13
TN	5	1H, 3H, 6H	5.3–6.4	4	4H, 5H	5.0–5.9	0		
DWT	3	3H	5.3–7.7	1	6H	6.0	1	6H	5.5
BY	5	5H, 7H	5.2–6.3	10	2H, 3H, 5H	5.4–7.1	14	2H, 3H, 5H	5.0–8.2
HW	9	1H, 2H, 4H	5.6–8.3	4	1H	6.5–35	3	1H	8.9–21
HI	1	2H	5.9	7	3H, 5H	5.2–20	4	3H	8.3–11
KS	3	3H, 5H	5.0–6.1	12	2H, 3H, 7H	6.0–11	10	2H, 3H	5.4–8.0
TKW	5	2H, 5H, 7H	5.2–6.3	0			3	2H, 5H, 7H	5.1–6.7
GY	2	1H	6.9–8.6	6	1H, 3H	5.2–18	5	1H, 3H	6.3–20
**PHYSIOLOGICAL**									
RWC	0			5	1H, 3H, 7H	5.1–6.1	1	1H	8.0
IPAR	3	2H, 3H	5.3–6.2	7	2H	8.0–17	6	2H	6.1–11
Fv/Fm	2	4H	5.4–5.7	4	1H, 3H	5.1–6.5	5	1H, 4H	5.8–8.8

PV (%), percentage of the phenotypic variation explained by SNP markers; NQ, number of significant QTLs; ChN, chromosome number.

Most of the QTLs were co-localized on chromosomes 2H and 3H; i.e., about 53% (30/57) and 62% (52/84) in the rain-fed and irrigation conditions, respectively. The chromosomes with lowest number of QTLs found were 4H and 6H, with two QTLs each, which accounted for 5.1–8.3% of the phenotypic variation. Only two QTLs were detected for grain yield in rain-fed condition, SNPs 2711-234 (1H) and 1923-265 (1H), which explained 7 and 8.6% of phenotypic variation, respectively, but importantly, they were stables across the two contrasting water regimes. Under full irrigation, six QTLs were detected for grain yield, SNPs 2711-234 (1H), 1923-265 (1H), ConsensusGBS0598-3 (3H), 9018-522 (3H), 4105-1417 (3H), 7045-950 (3H), and explained from 5.2 to 18%. Different to grain yield, the putative QTLs underlying biological yield were detected on chromosomes 5H and 7H, in the rain-fed condition, and on chromosomes 2H, 3H, and 5H in the fully irrigated condition.

All QTLs detected for BY, Fv/Fm, DWT, HI, IPAR, KS, PE, PH, SL, RWC, TKW, and TN were environment-specific. Although most QTL-trait associations were environment specific, some stable associations were also detected for HW, SNPs 2711-234 (1H) and 1923-265 (1H), and PL, SNPs 9282-205 (3H), 3965-353 (3H), 2335-1614 (3H), and 5212-1409 (7H).

Fifteen and ten SNPs, respectively, were associated with more than one trait in the fully irrigated and rain-fed conditions. Importantly, the SNPs 4105-1417, 7045-950, 9018-522, ConsensusGBS0598-3 (all on chromosome 3H) were concomitantly associated with grain yield, harvest index, and kernel per spike, in the fully irrigated condition, which accounted for 5.2–20% of the phenotypic variation. The mentioned SNPs ConsensusGBS0598-3, 7045-950, and 4105-1417 were also associated to biological yield, and the SNP 9018-522 with spike length. In both environmental conditions, grain yield and HW shared QTLs linked to the SNPs 1923-265 and 2711-234 (on chromosome 1H); both QTLs correspond to stable associations across environments.

QTLs with >15% of the phenotypic variation explained by SNP markers are shown in Table 5; these are relatively moderate (to major) QTLs detected in this RSCL population of barley. The results confirmed the presence of more QTLs with >15% in the fully irrigated (9) than the rain-fed condition (2), and the majority of these QTLs were environment-specific. In the fully irrigated condition, a relatively major QTL was detected underlying HW, i.e., SNP marker 1923-265 on chromosome 1H at 140 cM, which accounted for 35.3% of the total phenotypic variation. In the same genomic region, the SNP marker 2711-234 (on 1H at 139 cM) explained 27% of the phenotypic variation for HW. These QTLs were stable across the contrasting environmental conditions. Interestingly, the major locus 1923-265 (1H) associated with HW, was also detected for grain yield in both environmental conditions, which explained between 8.6 and 18.1% of the phenotypic variation, in the rain-fed and fully irrigated conditions, respectively. Finally, one genomic region on chromosome 4H, was moderately associated with plant height (i.e., SNPs ABC08009-1-2-304 and 954-1377) and was environment-specific in the rain-fed condition.

**Table 5:** **Relatively moderate (or major) QTLs detected in a RSCL population of barley (only QTLs with >15% of the phenotypic variation explained by SNP markers), evaluated under well-watered and rain-fed conditions by a mixed modeling approach**.

**Trait**	**SNP**	**ChN**	**Pos**	**PV(%)**	**Situation**
**FULLY IRRIGATED PLANTS**					
PL	3965-353	3H	176	15.1	Both environments
IPAR	8787-1459	2H	39	16.8	Environment-specific
HI	ConsensusGBS0598-3	3H	60	15.6	Environment-specific
HI	9018-522	3H	61	16.9	Environment-specific
HI	4105-1417	3H	64	20.3	Environment-specific
HI	7045-950	3H	67	15.4	Environment-specific
GY	1923-265	1H	140	18.1	Both environments
HW	2711-234	1H	139	26.9	Both environments
HW	1923-265	1H	140	35.3	Both environments
**RAIN-FED**					
PH	ABC08009-1-2-304	4H	180	22.2	Environment-specific
PH	954-1377	4H	182	22.2	Environment-specific
**COMBINED**					
PL	3965-353	3H	176	18.3	Both environments
GY	1923-265	1H	140	20.4	Both environments
HW	2711-234	1H	139	17.1	Both environments
HW	1923-265	1H	140	21.4	Both environments

PV(%), percentage of the phenotypic variation explained by SNP markers; ChN, chromosome number; Pos, SNP position in cM.

## Discussion

In this study, a RCSL population consisting of 137 lines was evaluated for 15 complex traits, including plant height, grain yield, and yield-related traits in two contrasting environment conditions. Severe allele frequency distortions was evidenced along the seven linkage groups in the RCSL population. By incorporating kinship into the model, as proposed by Malosetti et al. (2011) for populations that have undergone some selection resulting in a departure from Mendelian segregation ratios, we identified QTLs associated with key traits in two contrasting field conditions.

Environment-specific genomic regions were detected for the majority of the traits (12/15). These findings are consistent with the study conducted by Wang et al. (2014), in which most of the QTL for different traits varied between environments in a doubled haploid population of barley.

Most of the economically important traits in barley are inherited quantitatively. Plant height (PH), for instance, is under polygenic control, and represents one of the most important agronomic traits for barley (Wang et al., 2014; Zhou et al., 2015). In this study, the highest number of QTL was detected for plant height (20 QTL in both conditions) which were observed on chromosomes 2H, 3H, 4H, and 6H. Similarly, Honsdorf et al. (2014) found the highest number of associations for this trait, which were located on all chromosomes except 5H. Inostroza et al. (2009) found SSR-trait associations for PH on chromosomes 1H, 2H, 4H, 5H, 6H, and 7H, evidencing a genome-wide distribution. Interestingly, in the present study, a significant genomic region (explained 22% of phenotypic variance) on chromosome 4H, which comprises the SNPs ABC08009-1-2-304 and 954-1377, controls PH in barley under rain-fed conditions. The majority of the QTLs were detected on chromosomes 2H (4/7) under fully irrigated and 4H (5/7) under rain-fed condition. This result is partially consistent with Pasam et al. (2012), who found 32 associations with plant height with the majority located on chromosomes 2H and 3H. Malosetti et al. (2011) found significant SNP associations with PH on chromosomes 2H, 3H, 5H, and 7H, with two important QTLs on chromosomes 3H (127.1 cM) and 5H (69.3 cM), which are known to carry semi-dwarfing genes in barley (Malosetti et al., 2011; Wang et al., 2014). The use of semi-dwarf genes has greatly improved barley yields with controlled plant height being used to reduce yield loss arising from lodging and to increase the harvest index (Wang et al., 2014). Zhou et al. (2015) found a major QTL for plant height mapped at 105.5 cM on chromosome 3H, which had a LOD score of 13.01 and explained 44.5% of phenotypic variation. In this study, one QTL was detected at 197 cM on chromosome 3H (SNP 9610-1195), in the rain-fed condition, which explained 7.3% of phenotypic variation.

In the fully irrigated condition, a relatively major QTL was found for HW on chromosome 1H at 140 cM (SNP 1923-265), which explained 35.3% of phenotypic variation. This result is consistent with the study of Rode et al. (2012) who found three QTLs for HW on chromosome 1H, including the SNP 1923-265. In rain-fed condition, this SNP explained 8.6% of phenotypic variation for grain yield, and under fully irrigated condition, it explained 5.7 and 18.1% for RWC and GY, respectively. In contrast, Rode et al. (2012) did not find any SNP controlling HW associated with another related-trait. As expected, the SNP marker 2711-234 (in the same genomic region of SNP 1923-265 on chromosome 1H, at 139 cM) that explained 27% of the phenotypic variation for HW, was associated with grain yield in both environmental conditions, explaining 10 and 7% of phenotypic variation. The correlation coefficient between both traits was positively correlated (*r* = 0.48; *P* < 0.01; Table 3).

According to Naz et al. (2014), tiller number per plant is a major determinant of yield in crops like barley. In this study, the nine QTL detected for TN were located on all chromosomes except 2H and 7H, and all were environment-specific. There have also been conflicting reports on the QTL detection for TN and their chromosomal location in barley, although chromosomes 3H and 4H appear to be consistent (Elberse et al., 2004; Wang et al., 2010; Honsdorf et al., 2014). Naz et al. (2014) identified five QTL for TN on chromosomes 1H, 2H, 4H, and 5H; of which one QTL (located on 5H between 203.85 and 231.75 cM) accounted for 70.5% increase in TN. On the other hand, Honsdorf et al. (2014), for instance, found two QTL for this trait on chromosomes 3H and 4H.

Thousand kernel weight (TKW) is one of the major yield components having direct effect on the final yield (Pasam et al., 2012). In this study, five significant QTLs associated with TKW were found on chromosomes 2H (three QTLs), 5H (one QTL), and 7H (one QTL), under rain-fed condition, which explained between 5.2 and 6.3% of the phenotypic variation (Table 3). In contrast, Pasam et al. (2012) found 21 QTL associated with thousand grain weight, which were present on all chromosomes. Comadran et al. (2011) detected three QTL associated with TKW on chromosome 2H, and, similarly with our study, none of these associations accounted for more than 10% of the phenotypic variation; the largest effect was over 5% of the trait mean. Kalladan et al. (2013) found consistent QTL for TKW across the environments (stable QTL), which were mapped to all seven linkage groups except chromosomes 4H and 5H.

Two genomic region on chromosomes 1H and 3H were associated with grain yield under favorable conditions. These correspond to the SNP markers 2711-234 and 1923-265, on chromosome 1H, and ConsensusGBS0598-3 (60 cM), 9018-522 (61 cM), 4105-1417 (64 cM), and 7045-950 (67 cM) on chromosome 3H. Similarly, Rode et al. (2012) found two QTLs associated with grain yield on chromosome 3H, but at 42.1 cM (SNP 15141-288) and 169.3 cM (SNP ConsensusGBS0632-3). The two QTLs detected for grain yield in rain-fed conditions, SNPs 2711-234 (1H) and 1923-265 (1H), were stables across the two contrasting water regimes. This result is in accordance with the findings of Kalladan et al. (2013) who found altogether five stable QTL for yield; of them three were mapped to chromosome 1H: Moreover, the QTL explaining most of the phenotypic variations for yield were found on chromosome 1H and 2H. In contrast, seven QTLs were found in the Rode's study on chromosome 5H. Comadran et al. (2011) found three main QTL for grain yield located on chromosomes 2H and 7H. Inostroza et al. (2009) although using simple sequence repeats (SSRs) found that the yield QTLs are distributed throughout the genome, on chromosomes 1H, 2H, 3H, 5H, 6H, and 7H; similar to the findings of Mansour et al. (2014).

Importantly, Comadran et al. (2011) mentioned that co-localization of several QTL related to yield components traits suggest that major developmental loci may be linked to most of the associations in barley. In our study, some markers on chromosome 3H were concomitantly associated with biological and grain yield, harvest index, spike length, and kernel per spike, in the irrigated environment. Most of the correlation coefficients among these traits were positive and statistically different from zero (*P* < 0.01) varying from *r* = 0.24 to 0.64. In both environmental conditions, grain yield and HW (correlation coefficient *r* = 0.48, *P* < 0.01) shared two QTLs located on chromosome 1H (SNPs 1923-265 and 2711-234), both QTLs correspond to stable associations across environments.

In this study, eight putative QTLs underlying harvest index were identified on chromosomes 2H, 3H, and 5H. Most of them were localized on chromosome 3H (6/8) under irrigation conditions. Only one significant QTL (SNP 5880-2547) controlling HI was found on 2H under rain-fed conditions, which explained 6% of phenotypic variation. This QTL controlling HI was also concomitantly associated with thousand kernel weight, in rain-fed (explained 6%); the correlation coefficient between these traits was statistically different from zero *r* = 0.2, *P* < 0.05. Comadran et al. (2011) detected three QTL for HI on chromosomes 1H, 2H, and 3H, with the most significant being located on chromosome 2H, in the same region as QTL for heading date and yield.

Fan et al. (2015) found that the physiological trait RWC had a very close correlation (*r* = 0.73, *P* < 0.01) with drought tolerance in barley; in fact, one QTL for RWC was identified on chromosome 2H and it explained 44.3% of phenotypic variation. In the current study, no QTL controlling RWC was identified in rain-fed, and only a small effect was identified on 1H, 3H, and 7H accounting for around 5% of the phenotypic variation. This was expected considering that there was no significant difference in RWC between rain-fed and irrigated environments. Li et al. (2013) carried out a meta-analysis of QTL associated with tolerance to abiotic stresses in barley, identifying MetaQTL for RCW under abiotic stress on H1, H2, H5, H5, and H7. Wójcik-Jagła et al. (2013) in a comparative QTL analysis of early short-time drought tolerance in Polish fodder and malting spring barleys, found 18 QTLs for nine physiological traits on all chromosomes except 1H in malting barley and 15 QTLs for five physiological traits on chromosomes 2H, 4H, 5H, and 6H in fodder barley.

Fluorescence parameters for dark adapted flag leaves (Fo, Fm, Fv, Fv/Fm) of 194 recombinant inbred lines (RILs), developed from the cross between the cultivar “Arta” and *H spontaneum* 41-1, measured under well-watered and drought stress conditions, showed significant differences among RILs but no differences between water regimes (Guo et al., 2008). Using SSRs and AFLPs markers they were able to identified nine and five QTLs, under well-watered and drought stress conditions, respectively; a QTL for Fv/Fm [i.e., (Fm – Fo)/Fm], which explained 15% of the phenotypic variance, was identified on chromosome 2H at 116 cM in the linkage map under drought stress. In our study two QTLs for quantum yield of PSII on chromosome 4H were identify under rain-fed condition and five in the combined analysis (Table 4). In the study of Wójcik-Jagła et al. (2013) one major QTL related to photochemical quenching of chlorophyll fluorescence was located on chromosome 4H in fodder barley. In an advanced backcross quantitative trait locus (AB-QTL) analysis performed by Sayed et al. (2012) to elucidate genetic mechanisms controlling proline content (PC) and leaf wilting (WS) in barley under drought stress conditions, QTL for WS were localized on chromosome 1H, 2H, 3H, and 4H. Among these, QWS.S42.1H and QWS.S42.4H were associated to decrease in WS due to the introgression of exotic alleles. QTL for PC were localized on chromosome 3H, 4H, 5H, and 6H. QTL effects on 3H, 4H, and 6H were responsible to heighten PC due to the preeminence of elite alleles over the exotic alleles which ranged from 26 to 43%.

The results are in agreement with the findings of Malosetti et al. (2011) who screened a population of 161 inbred lines of barley with 1536 SNPs, which were used for gene and QTL detection. The model incorporating kinship, co-ancestry information, was consistently superior to the one without kinship (according to the Akaike information criterion), similarly with this study. Importantly, Malosetti et al. (2011) showed that ignoring this type of information results in an unrealistically high number of marker–trait associations, without providing clear conclusions about QTL locations. In this work, a large number of spurious QTLs were detected when the genetic covariance matrix is ignored in the mixed model (444 and 460 false-positives for all traits studied in the rain-fed and full irrigation conditions, respectively) confirming that ignoring this type of genetic relatedness will increase the rate of false-positives. As with the study carried out by Malosetti et al. (2011) we highlight the importance of the inclusion of kinship information when detecting QTL in populations that have undergone some process of selection. This research provides useful information for MAS programs in areas where drought is a significant constraint. As drought stress tolerance has become an important goal, this analysis of QTL identified significant genomic regions that can be used for breeding purposes.

## Author contributions

FM and YQ made the QTLs analysis and FM wrote the first manuscript; IM was responsible of the field trial and agronomic trait determination; JR and RW were responsible of the research grants CGIAR-GCP Challenge and performed the SNPs analysis; AD performed the physiological evaluations and is the leader of the drought tolerance studies of barley in Chile. All the authors contributed to the final manuscript.

### Conflict of interest statement

The authors declare that the research was conducted in the absence of any commercial or financial relationships that could be construed as a potential conflict of interest.
