# Supplementary material for: SNP-Based QTL Mapping of 15 Complex Traits in Barley under Rain-Fed and Well-Watered Conditions by a Mixed Modeling Approach
Source: Front Plant Sci. 2016 Jun 27;7:909. doi: 10.3389/fpls.2016.00909 (PMC4921488; doi:10.3389/fpls.2016.00909)
Supplement: Supplementary file 1 [file Table1.DOCX]

Table S1. Monthly minimum (T min) and maximum (T max) temperatures and precipitation (PP) at Santa Rosa in 2008.

|  | J | F | M | A | M | J | J | A | S | O | N | D |
| --- | --- | --- | --- | --- | --- | --- | --- | --- | --- | --- | --- | --- |
| T min (ºC) | 30.2 | 31.0 | 27.3 | 19.7 | 16.0 | 12.0 | 12.1 | 13.4 | 18.8 | 21.1 | 25.9 | 28.1 |
| T max (°C) | 12.7 | 12.1 | 9.0 | 6.3 | 5.3 | 4.6 | 6.5 | 4.7 | 5.3 | 7.0 | 9.5 | 11.6 |
| PP (mm) | 2.6 | 0.0 | 1.6 | 68.1 | 430.5 | 100.1 | 172.2 | 199.4 | 41.9 | 29.3 | 16.9 | 13.0 |
| ET (mm) | 274.7 | 247.0 | 173.4 | 57.6 | 24.2 | 1.5 | 1.1 | 2.9 | 55.8 | 108.8 | 189.2 | 226.1 |
