# Supplementary material for: SNP-Based QTL Mapping of 15 Complex Traits in Barley under Rain-Fed and Well-Watered Conditions by a Mixed Modeling Approach
Source: Front Plant Sci. 2016 Jun 27;7:909. doi: 10.3389/fpls.2016.00909 (PMC4921488; doi:10.3389/fpls.2016.00909)
Supplement: Supplementary file 2 [file Table2.docx]

Supplementary Table S2. Goodness of fit of the mixed model without kinship information (S model) and the model that includes the kinship information (K model).

| Trait | Rain fed | | | | Fully irrigated | | | | Combined | | | |
| --- | --- | --- | --- | --- | --- | --- | --- | --- | --- | --- | --- | --- |
|  | S model | | K model | | S model | | K model | | S model | | K model | |
|  | LL | BIC | LL | BIC | LL | BIC | LL | BIC | LL | BIC | LL | BIC |
| PL | 644 | 654 | 597 | 612 | 660 | 670 | 635 | 650 | 617 | 627 | 562 | 577 |
| PE | -12 | -2 | -18 | -3 | 82 | 92 | 80 | 95 | -19 | -9 | -23 | -8 |
| SL | 400 | 409 | 389 | 404 | 357 | 366 | 315 | 329 | 338 | 347 | 304 | 319 |
| PH | 885 | 894 | 808 | 823 | 937 | 947 | 861 | 876 | 883 | 892 | 782 | 797 |
| TN | 1519 | 1528 | 1504 | 1519 | 1703 | 1713 | 1691 | 1706 | 1585 | 1594 | 1565 | 1580 |
| DWT | 1419 | 1429 | 1419 | 1434 | 1553 | 1563 | 1553 | 1568 | 1396 | 1406 | 1396 | 1411 |
| BY | 1685 | 1695 | 1682 | 1697 | 1856 | 1865 | 1848 | 1863 | 1718 | 1728 | 1707 | 1722 |
| HW | 546 | 556 | 542 | 556 | 550 | 560 | 516 | 531 | 547 | 557 | 525 | 540 |
| HI | -330 | -320 | -357 | -342 | -398 | -388 | -437 | -422 | -416 | -406 | -453 | -438 |
| KS | 666 | 676 | 651 | 665 | 638 | 648 | 573 | 588 | 608 | 618 | 558 | 573 |
| TKW | 836 | 846 | 808 | 822 | 878 | 887 | 874 | 889 | 782 | 792 | 765 | 780 |
| GY | 111 | 121 | 73 | 88 | 453 | 462 | 405 | 419 | 305 | 315 | 249 | 264 |
| RWC | 838 | 848 | 834 | 849 | 904 | 913 | 900 | 915 | 791 | 801 | 788 | 802 |
| IPAR | 890 | 900 | 882 | 897 | 934 | 944 | 926 | 940 | 842 | 852 | 828 | 843 |
| Fv/Fm | -385 | -375 | -385 | -371 | -553 | -543 | -558 | -544 | -545 | -535 | -545 | -531 |

LL: -2 Ln (likelihood); BIC: Bayesian Information Criterion (smaller is better)
